# Ganglioside and Non-ganglioside Mediated Host Responses to the Mouse Polyomavirus

**DOI:** 10.1371/journal.ppat.1005175

**Published:** 2015-10-16

**Authors:** John You, Samantha D. O’Hara, Palanivel Velupillai, Sherry Castle, Steven Levery, Robert L. Garcea, Thomas Benjamin

**Affiliations:** 1 Department of Microbiology and Immunobiology, Harvard Medical School, Boston, Massachusetts, United States of America; 2 BioFrontiers Institute and the Department of Molecular, Cellular and Developmental Biology, University of Colorado, Boulder, Colorado, United States of America; 3 Department of Chemistry, University of New Hampshire, Durham, New Hampshire, United States of America; Penn State University School of Medicine, UNITED STATES

## Abstract

Gangliosides serve as receptors for internalization and infection by members of the polyomavirus family. Specificity is determined by recognition of carbohydrate moieties on the ganglioside by the major viral capsid protein VP1. For the mouse polyomavirus (MuPyV), gangliosides with terminal sialic acids in specific linkages are essential. Although many biochemical and cell culture experiments have implicated gangliosides as MuPyV receptions, the role of gangliosides in the MuPyV-infected mouse has not been investigated. Here we report results of studies using ganglioside-deficient mice and derived cell lines. Knockout mice lacking complex gangliosides were completely resistant to the cytolytic and pathogenic effects of the virus. Embryo fibroblasts from these mice were likewise resistant to infection, and supplementation with specific gangliosides restored infectibility. Although lacking receptors for viral infection, cells from ganglioside-deficient mice retained the ability to respond to the virus. Ganglioside-deficient fibroblasts responded rapidly to virus exposure with a transient induction of *c-fos* as an early manifestation of a mitogenic response. Additionally, splenocytes from ganglioside-deficient mice responded to MuPyV by secretion of IL-12, previously recognized as a key mediator of the innate immune response. Thus, while gangliosides are essential for infection in the animal, gangliosides are not required for mitogenic responses and innate immune responses to the virus.

## Author Summary

Biological and structural studies have combined to give a detailed understanding of how the mouse polyomavirus binds to sialyloligosaccharides, how polymorphisms in the sialic acid binding pocket of the major virus capsid protein constitute important determinants of pathogenicity, and how gangliosides function as receptors for cell entry and infection by the virus. We used mice with knockouts in defined ganglioside biosynthetic pathways to determine whether gangliosides alone suffice to mediate lethal infection in the intact host and whether non-gangliosides are also recognized by the virus and utilized for important physiological responses. We confirmed the requirement of specific gangliosides for infection and determined that not all gangliosides that bind *in vitro* serve as receptors *in vivo*. Results also revealed two physiologically important responses that do not require MuPyV-ganglioside interactions: i) rapid induction of *c-fos* in fibroblasts as an early step in cell cycle progression on which the virus depends for its own replication, and ii). activation of cytokine secretion by antigen presenting cells as a critical innate immune response to the virus. We infer that these responses are mediated by non-ganglioside receptors bearing sialic acid. These results serve to illustrate the multiplicity of MuPyV receptors and the complexity of virus-cell surface interactions.

## Introduction

The *Polyomaviridae* comprise an expanding family of viruses of human, non-human primate and rodent origin as well as several avian species [1]. These small non-enveloped icosahedral DNA viruses are similar in their structural and genetic organization. Studies in cell culture with several members of the group have demonstrated that gangliosides serve as necessary receptors for infection. Initial studies showed mouse polyomavirus (MuPyV) binding to specific gangliosides in the plasma membrane leads to internalization and transport via endolysosomes to the endoplasmic reticulum [2]. There the virus is thought to undergo partial disassembly followed by translocation to the cytosol and nuclear entry. Steps of virus disassembly leading to export from the endoplasmic reticulum are partially understood [3–8].

Gangliosides are sialic acid containing glycosphingolipids that are ubiquitously expressed. Gangliosides are anchored in the outer leaflet of the plasma membrane by a ceramide tail with their sialylated oligosaccharide portion (glycan) facing extracellularly. The level of gangliosides required for infection by MuPyV are controlled in part through regulation of a sialidase activity by tyrosine kinases of the Abl family [9]. Binding specificity among the polyomaviruses is based on recognition of the glycan by the major viral capsid protein VP1. High-resolution structural and biochemical studies have revealed details of how recognition of sialic acids in various linkages occur with different polyomaviruses [10–17]. MuPyV binds to oligosaccharides carrying terminal sialic acids in specific linkages found in several gangliosides. Studies with different strains of MuPyV have shown how differences in glycan recognition underlie biological properties. MuPyV has also been shown to bind to the α4β1 integrin. Mutagenesis of the integrin binding site on VP1 decreases infectivity by 50%, suggesting that α4β1 may serve as a ‘co-receptor’ mediating a post-attachment step of infection [18, 19].

The outcome of infection by MuPyV depends on the genetic background of both virus and host. Inbred strains of mice have been used to identify host determinants that underlie susceptibility or resistance to the virus [20]. Strains of MuPyV differing widely in pathogenicity owe their differences to polymorphisms in VP1 that allow the virus to discriminate among different oligosaccharides or that affect avidity of binding to sialic acid [10, 11, 21–25]. High-resolution structural studies of complexes between recombinant VP1s of several MuPyV strains and various glycans have extended and refined our understanding of receptor interactions [26]. Here we utilize mice with knockouts in ganglioside biosynthetic pathways to investigate the importance of specific gangliosides for infection and to determine whether gangliosides are essential for other host-responses such as mitogenic gene induction and innate immunity.

## Results

### Disruptions of ganglioside biosynthetic pathways in knockout mice

**Fig 1:**
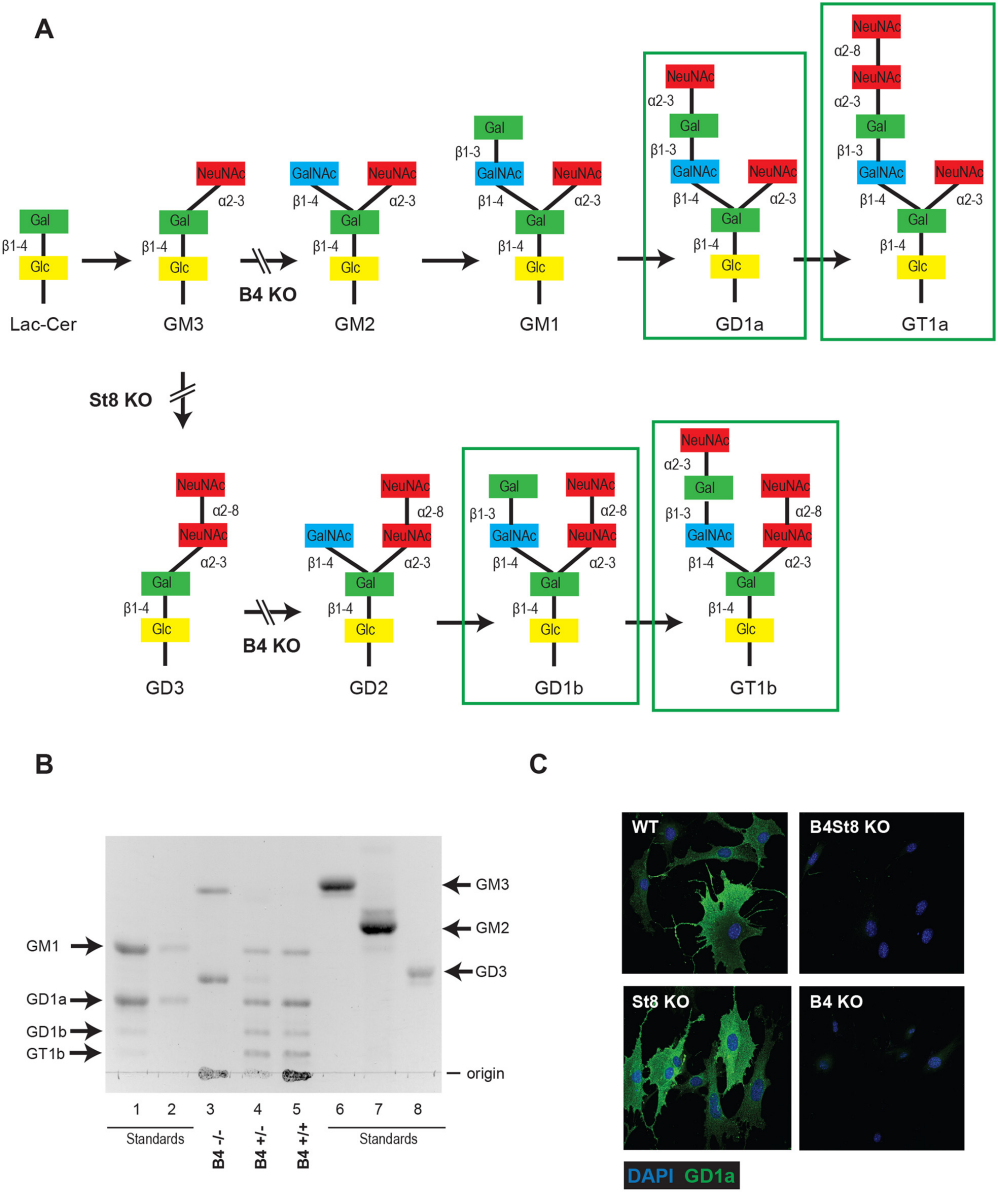
(A) B4 and St8 knockouts in pathways of ganglioside biosynthesis. Gangliosides previously shown or shown here to function as MuPyV receptors are outlined. Modified from U. Neu and T. Stehle. (B) High performance TLC on acidic lipid fractions from kidneys of wild-type and B4 knockout mice. Lane 1 –standards for GM1, GD1a, GD1b and GT1b; lane 2 –standards for GM1 and GD1a loaded at 50% volume of lane 1; lane 3 –B4 -/-; lane 4—B4 +/-; lane 5 –B4 +/+; lane 6 –GM3 standard; lane 7 –GM2 standard; lane 8 –GD3 standard. (C) St8 KO MEFs retain a-series gangliosides. GD1a staining of WT and St8 KO MEFs show that both cell lines express GD1a. B4 KO and B4St8 KO MEFs do not express GD1a.

Previous studies have used ganglioside-deficient cell lines (*i*.*e*., rat glioma C6 cells, R- mouse cells) to evaluate the importance of ganglioside receptors for MuPyV infection [2, 9]. These cell lines are often from a heterologous-host for MuPyV, and are not genetically defined. Thus, we generated ganglioside-deficient mice with known ganglioside composition to clearly identify the role of specific gangliosides in MuPyV infection. The B4 KO mouse is blocked in a β1–4 GalNAc transferase (GM2/GD2 synthase) and is expected to lack the previously identified MuPyV ganglioside receptors, GD1a and GT1b, while maintaining expression of GM3 and GD3 (Fig 1A). We validated the ganglioside composition in B4 KO mice by analyzing total acidic lipid fractions from kidneys, a major site of replication and tissue destruction by MuPyV. High performance thin layer chromatography of kidney lipid fractions from uninfected wild type and B4 KO mice confirmed that only GD3 and its precursor GM3 are made in B4 KO mice (Fig 1B, lane 3). B4 heterozygous (+/-) mice showed decreased levels of gangliosides compared to wild-type mice (Fig 1B, lane 4). Immunofluorescence staining also showed that B4 KO mice lack a-series gangliosides such as GD1a (Fig 1C).

GD3 has been shown to bind pentamers of MuPyV VP1 in an *in vitro* screen, but its function as a receptor has not been evaluated. To determine the possible role of GD3 in MuPyV infection, St8 mice lacking -2,8 sialyltransferase (GD3 synthase) were generated. St8 mice cannot synthesize b-series gangliosides, including GD3 and its derivatives (Fig 1A), but retain a-series gangliosides, such as GD1a as verified by immunofluorescence staining with a GD1a antibody (Fig 1C). Thus, the B4St8 double KO mouse is expected to synthesize only GM3, which was previously shown to be unable to bind or mediate infection by MuPyV [2]. Protein glycosylation pathways are expected to be unaltered in these ganglioside-deficient mice.

### B4 KO mice survive infection by a lethal strain of MuPyV

**Fig 2:**
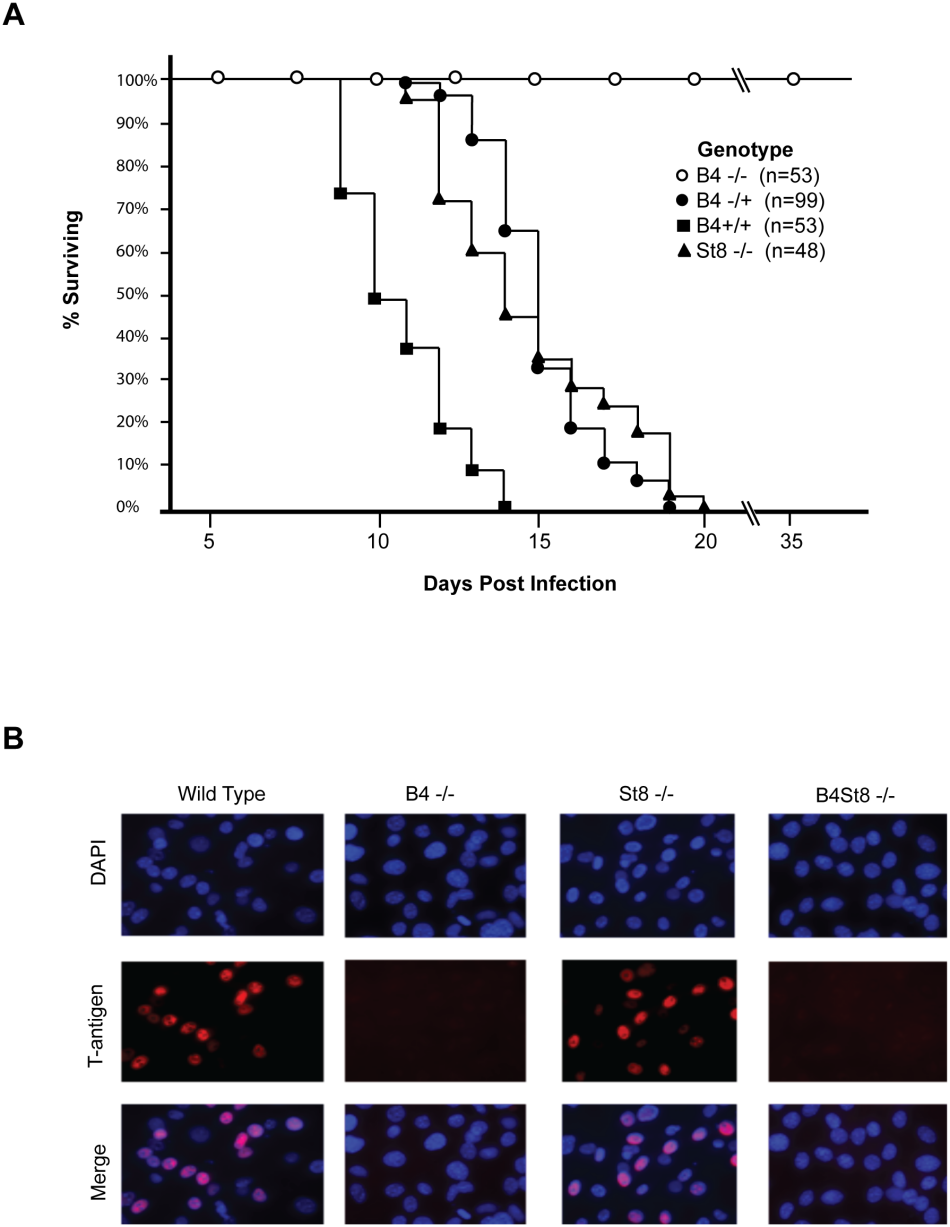
Characterization of St8 and B4 KO mice. A. Kaplan-Meier survival curves for wild-type and ganglioside-knockout mice. Newborn mice were inoculated intraperitoneally with ~10^6^ PFU of the LID strain of virus and followed using death as an endpoint. B. Susceptibility of wild-type, ST8, and B4 MEFs to infection. Large T-antigen immunofluorescence of MuPyV-infected mouse embryo fibroblasts from wild-type and ganglioside-deficient mice. Cells were infected with the RA virus at an MOI of 1–2 PFU/cell.

The LID strain of MuPyV induces a rapidly lethal infection of newborn mice of most backgrounds [23, 27]. Mice typically succumb within a few weeks due to a widely disseminated infection with extensive destruction of the kidney and other vital tissues. LID owes its virulence to an amino acid substitution in the major capsid protein VP1 that reduces hydrophobic interactions with the sialic acid ring. This lower avidity binding of virus to cells facilitates release from cell debris and promotes virus spread. To establish the importance of gangliosides in mediating this infection, newborn mice from a cross of heterozygous B4 KO mice were inoculated with LID. Mice were followed daily and death was used as an endpoint (Fig 2A). Genotyping was carried out retrospectively, *i*.*e*., at time of death or at the end of the experiment.

Wild-type mice (B4 +/+) all succumbed within 14 days, as expected. Homozygous knockout mice (B4 -/-) all survived and showed no overt signs of illness at 35 days post infection when the experiment was terminated. Heterozygous mice (B4 +/-) also succumbed, though with a delay compared to wild-type mice. These mice express slightly decreased levels of gangliosides compared to wild-type mice (Fig 1B). A single copy of the GM2/GD2 synthase gene targeted in the B4 KO mouse thus sufficed to confer susceptibility. The extended survival of B4 heterozygotes is consistent with a gene dosage effect, whereby overall levels of enzyme activity (*i*.*e*., ganglioside synthesis) correlate inversely with mean survival time. Results with homozygous St8 KO mice were also consistent with this view. These mice (St8 -/-) all succumbed, but like B4 +/-, survived longer than wild-type mice (Fig 2A). The St8 mice retain GD1a and other a-series gangliosides (Fig 1A and 1C) indicating that these receptors are sufficient for lethal LID infection in the absence of GT1b and other b-series gangliosides. Thus, MuPyV infection is delayed by either decreased ganglioside diversity or decreased abundance of complex gangliosides. These results establish the importance of complex gangliosides lacking in the B4 KO mouse for mediating infection, and confirm for the first time that specific gangliosides are required for virus infection *in vivo*. These results establish the importance of complex gangliosides lacking in the B4 KO mouse for mediating infection by the LID strain of MuPyV. They confirm for the first time that specific gangliosides are required for virus infection *in vivo*.

### Susceptibility of ganglioside-deficient cells to MuPyV infection

Mouse embryo fibroblast cultures (MEFs) were established from wild-type and ganglioside-deficient mice. These cells were used to access the degree of resistance and the roles of specific gangliosides in mediating infection. MEFs were first infected with the standard laboratory small plaque strain RA at a multiplicity of infection (MOI) of 1–2 plaque forming units (PFU) per cell. Cells were fixed at 24 hrs post-infection and stained with anti-T (tumor) antigen antibody to determine the number of infected cells expressing the nuclear large T protein (Fig 2B). B4 KO MEFs were resistant while St8 KO MEFs were susceptible to infection. The B4St8 double KO cells, like those from B4, were resistant. Two independently derived lines of MEFs of each genotype were tested and showed the same results. B4 KO and B4St8 KO MEFs produced normal yields of virus following transfection with MuPyV DNA or supplementation with gangliosides, confirming that their resistance is due to a block in cell entry prior to uncoating of the virus and not to a block in the virus replication cycle *per se*. The resistance of B4 and B4St8 KO MEFs extended equally to small plaque (RA) and large plaque (PTA) strains (Table 1). When the MOI was increased from 1–2 PFU/cell to 5–10 PFU/cell, a few percent of the B4 KO MEFs were infected. In contrast, B4St8 KO MEFs remained completely resistant at the higher MOI (see further below).

**Table 1:** Susceptibility of wild-type and ganglioside deficient MEFs to small plaque and large plaque polyomavirus strains.

	Cells			
Viruses(PFU/cell)	WT	B4	ST8	B4ST8
**RA (~1–2)**	228/504 = 45.2%	0/616 = 0	233/533 = 43.7%	0/512 = 0
**RA (~5–10)**	463/506 = 91.5%	15/602 = 2.5%	500/573 = 87.2%	0/523 = 0
**PTA (~1–2)**	215/517 = 40.8%	0/509 = 0	201/532 = 37.8%	0/542 = 0
**PTA (~5–10)**	467/553 = 84.4%	9/519 = 1.7%	431/534 = 80.7%	0/523 = 0

### GD3 does not function as a receptor in mice despite binding VP1 *in vitro*

B4 KO mice express only GD3 and GM3 and are not susceptible to lethal LID infection (Fig 2A). GM3 was previously shown to be unable to bind virus based on a flotation assay using ganglioside-supplemented liposomes [2]. However, GD3 has been identified in a glycan array screen as a strong binder of recombinant VP1 pentamers. A co-crystal structure of recombinant VP1 pentamers with the GD3 glycan has been determined [26]. GD3 also emerges as a possible receptor based on the observation that at high MOI a small percent of B4 KO, but not B4St8 double KO MEFs, become infected (Table 1). B4 KO MEFs synthesize GD3 while B4St8 MEFs do not (Fig 1A). Supplementation experiments were carried out to directly test whether GD3 is a functional receptor. B4St8 KO MEFs were incubated for 2 hrs in media with increasing concentrations of GD3, then washed and infected with RA at a MOI of 5 to10 PFU/cell. Resistance was overcome, but only a few percent of cells were capable of being infected even at the highest concentrations of GD3 tested (Table 2). Thus, under conditions of supplementation with high concentrations of GD3 and a high virus input, some virus particles apparently engage enough of the ganglioside to allow cell entry and infection. Although these conditions may exist *in vivo*, they do not lead to lethal infection in B4 KO mice (Fig 2A). GD3 accumulates to high levels in kidneys of B4 KO mice (see Fig 1B, lane 3). Despite the high endogenous levels of GD3, B4 KO mice are resistant to infection (Fig 2A). We conclude that GD3 does not serve as an efficient functional receptor *in vivo* despite its ability to bind the viral capsid subunits *in vitro*.

**Table 2:** GD3 Supplementation of B4ST8 MEFs.

		GD3 Concentration			
Cells	RA (PFU/cell)	None	50 μM	100 μM	200 μM
WT	~5–10	528/567 = 93.1%	n/a	n/a	n/a
B4St8	~5–10	0/523 = 0%	3/521 = 0.6%	7/543 = 1.3%	10/514 = 2.0%

### Supplementation of GT1a and GD1b restores infection of B4St8 MEFs

**Fig 3:**
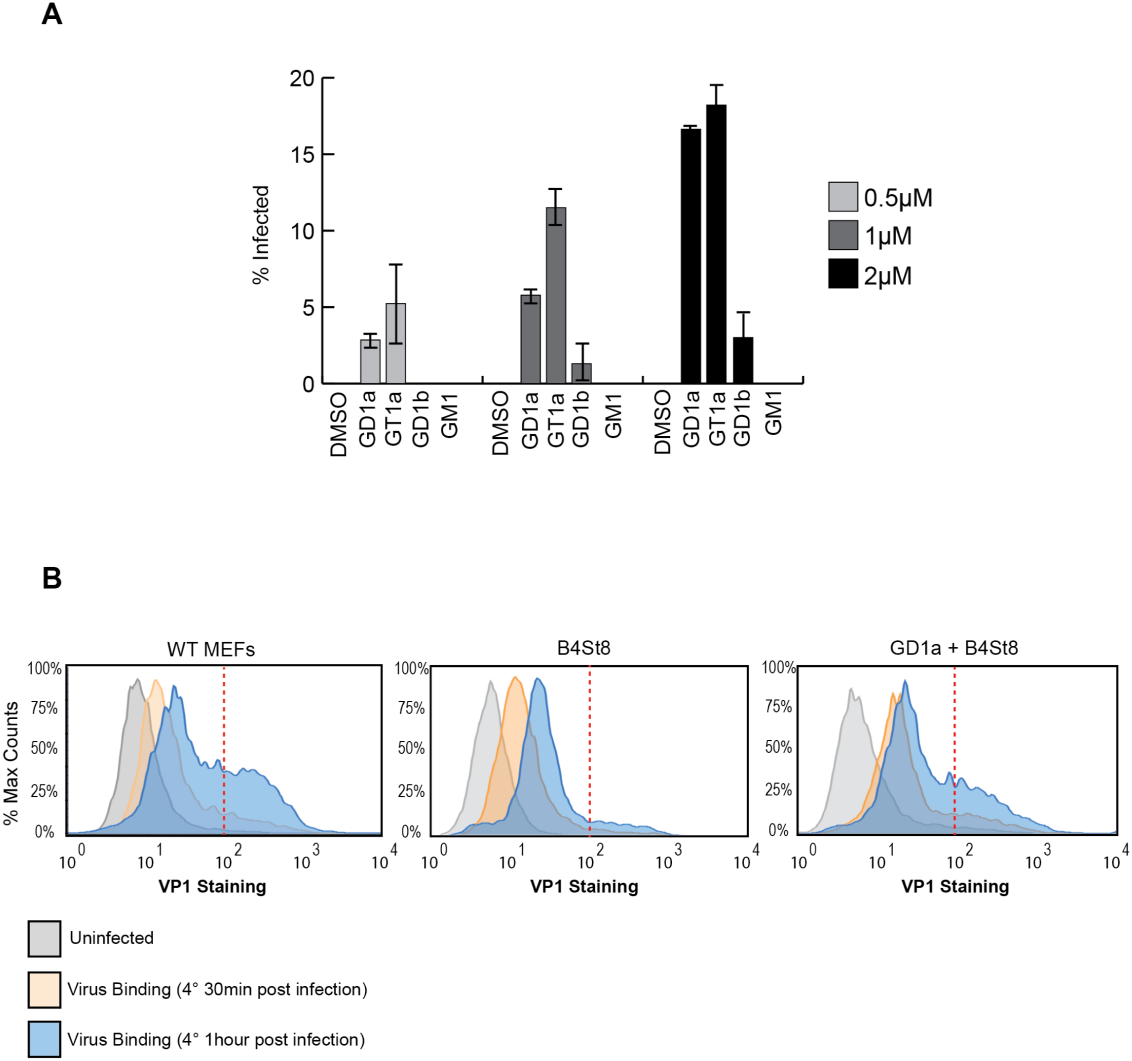
A. GT1a and GD1b restore infectibility to B4St8 MEFs. GT1a, GD1a, and GD1b supplementation of B4St8 KO MEFs rescued RA infection in a dose responsive manner (0.5μM to 2μM). GM1 supplementation did not rescue infection of B4St8 KO MEFs. B. Virus binding to wild-type, B4St8, and GD1a-supplemented B4St8 MEFs. WT, B4St8, and GD1a-supplemented B4St8 KO MEFs were assayed for MuPyV binding by flow cytometry. Cells were starved overnight in serum free media with or without 5μM GD1a. Cells were then infected with MuPyV (NG59RA, MOI 10) at 4°C. After 30 mins at 4°C, bound virus was detected by VP1 staining. WT, B4St8, and GD1a-supplemented B4ST8 KO MEFs had a 6 to 8 fold increase in VP1 staining compared to uninfected cells (shown in grey). GD1a-supplemented B4St8 KO MEFs showed a similar VP1 binding pattern as WT MEFs and B4St8 KO MEFs showed lower levels of VP1 accumulation on the cell surface. The x-axis is VP1 staining and the y-axis is normalized cell counts, each sample contained >10,000 cells.

GD1a and GT1b have previously been shown to confer susceptibility to MuPyV infection when added to ganglioside-deficient rat and mouse cell lines [2, 28, 29]. We sought to extend these results using our B4St8 KO cells, which are genetically defined and have known ganglioside composition. GD1a was used as a positive control, and GM1, the SV40 receptor, as a negative control to confirm previous results. We then tested the ability of additional gangliosides, GT1a and GD1b, to confer susceptibility using the RA strain of virus. GT1a had been suggested as a possible receptor based on co-crystallization with MuPyV VP1 [26]. Cells were pre-incubated with 0.5 to 2.0 μM gangliosides in serum-free medium for 16 hrs, then infected and scored for T-antigen expression 24 hrs post-infection. GT1a conferred infectibility slightly more efficiently than GD1a, a result consistent with *in vitro* affinity studies [26], and GD1b conferred low levels of infectibility, much less efficiently than GT1a or GD1a (Fig 3A).

### Gangliosides are required for high levels of virus accumulation on the cell surface

The VP1 binding pocket of MuPyV is thought to accommodate both glycolipid (ganglioside) and glycoprotein binding [10,11]. Thus, we investigated the dynamics and levels of cell surface binding of virus in the presence or absence of ganglioside receptors. Wild-type, B4St8 KO, and GD1a-supplemented B4St8 KO MEFs were infected (RA MuPyV, 10 PFU/cell) at 4°C followed by fixation and staining for cell surface VP1 using a VP1 antibody. Virus binding to wild-type MEFs at 4°C was time dependent with two cell populations at 1 hr post virus addition: a cell population with low virus accumulation (<10^2^ VP1 staining) and a cell population with high virus accumulation (>10^2^ VP1 staining) (Fig 3B). B4St8 KO MEFs were also bound by virus in a time dependent manner; however, these cells displayed only low virus accumulation after 1 hr at 4°C (<10^2^ VP1 staining) (Fig 3B). Supplementation of B4St8 KO MEFs with 5μM GD1a prior to virus addition restored virus accumulation on the cell surface after 1 hr at 4°C (>10^2^ VP1 staining) (Fig 3B). These data indicate that although virus binds cells in the absence of ganglioside receptors, the dynamics of binding are changed. Gangliosides result in high levels of virus accumulation on the cell surface, whereas alternative interactions result in lower levels of overall virus binding.

### Virus endocytosis occurs in ganglioside-deficient fibroblasts

**Fig 4:**
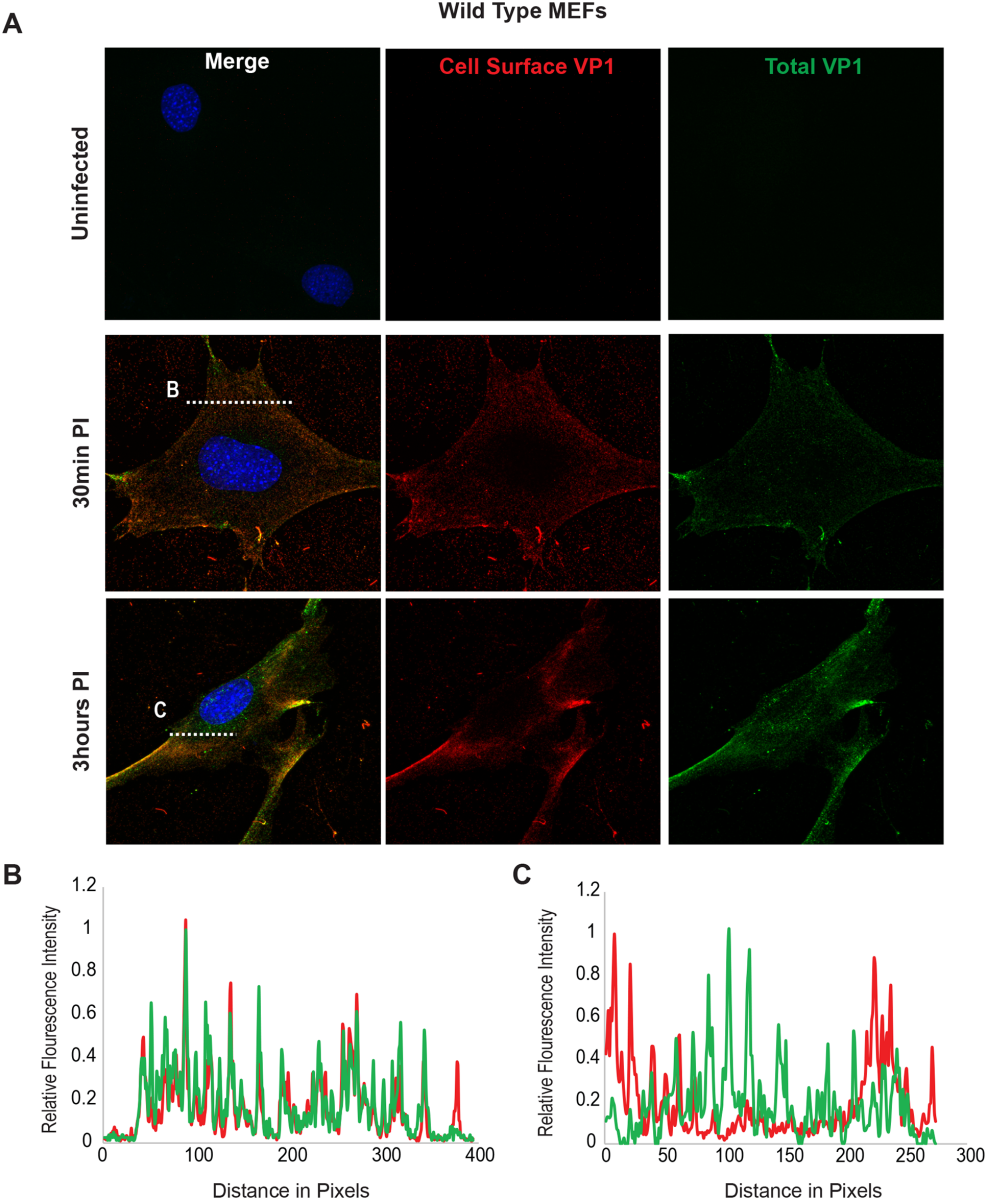
Virus Internalization in Wild-Type MEFs. Wild-type MEFs were infected with MuPyV and then fixed at the indicated times post infection (30 min, 3hrs). **(A)** Slides were stained for cell surface VP1 (red), and then permeabilized and stained for total VP1, showing both cell surface and intracellular VP1 (green). **(B)** At 30 mins post-infection line scan analysis shows that MEFs exhibit similar staining for cell surface and total VP1, indicating minimal virus internalization. **(C)** At 3 hrs post-infection line scan analysis shows that MEFs exhibit abundant intracellular VP1 staining (green only), indicating internalized virus.

**Fig 5:**
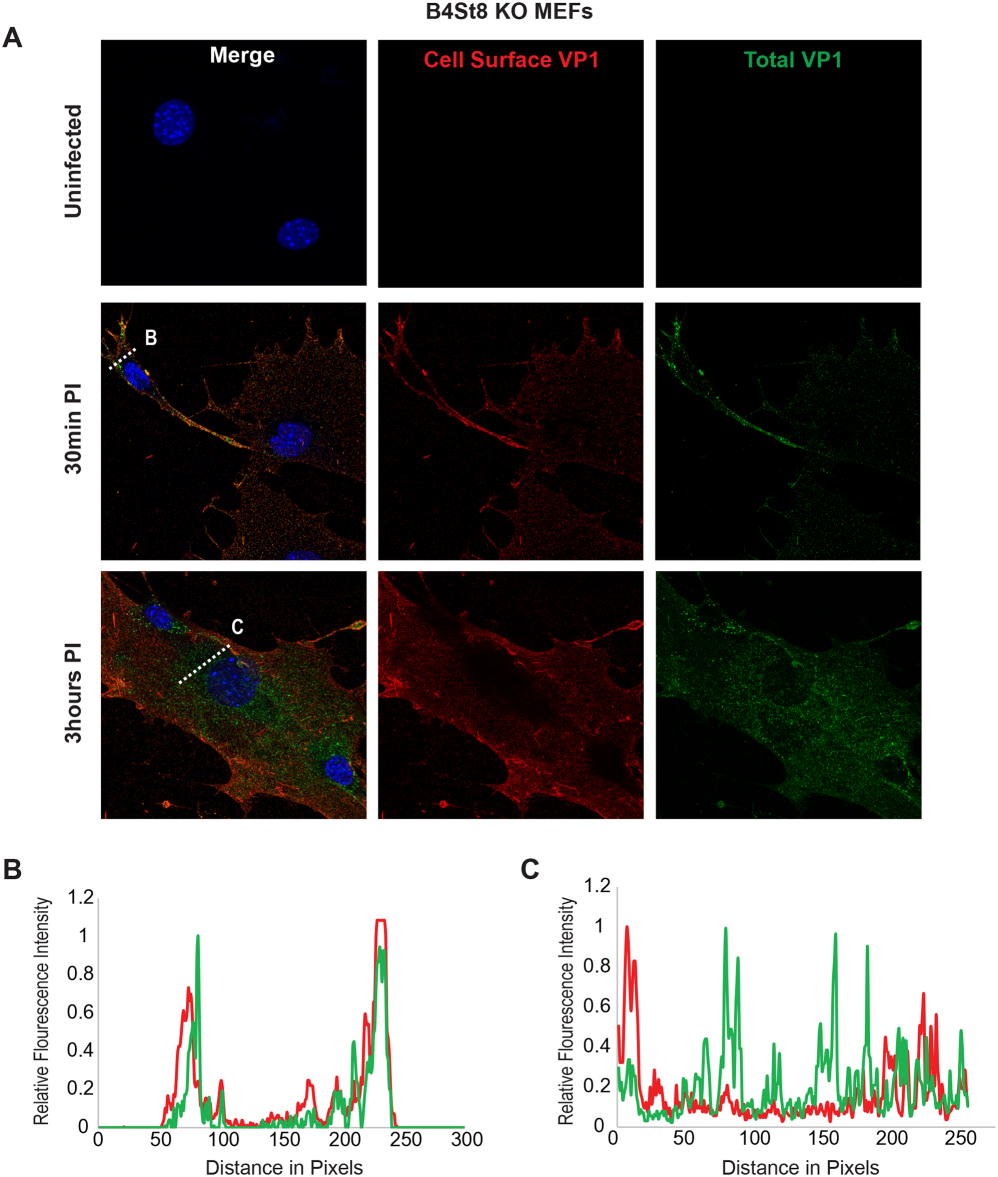
Virus Internalization in B4St8 KO MEFs. B4St8 knock out MEFs were infected with MuPyV and then fixed at the indicated times post infection (30 min, 3 hrs). **(A)** Slides were stained for cell surface VP1 (red), and then permeabilized and stained for total VP1, showing both cell surface and intracellular VP1 (green). **(B)** At 30 mins post-infection line scan analysis shows that B4St8 MEFs exhibit similar staining for cell surface and total VP1, indicating minimal virus internalization. **(C)** At 3 hrs post-infection line scan analysis shows that B4St8 KO MEFs exhibit abundant intracellular VP1 staining (green only), indicating internalized virus.

Using the B4St8 ganglioside KO MEFs we determined whether gangliosides are required for virus entry. Wild-type MEFs and B4St8 KO MEFs were infected with MuPyV (RA, 50 PFU/cell) and then fixed at the indicated times post-infection (30 min, 3 hrs). At 30 mins post-infection in wild-type MEFs line scan analysis showed that MEFs exhibit similar staining for cell surface (shown in red) and total VP1 (shown in green), indicating minimal virus internalization at this early time (Fig 4B). Similar results were seen in B4St8 KO MEFs at 30 min post infection (Fig 5B). At 3 hrs post-infection in wild-type MEFs line scan analysis showed that MEFs had abundant intracellular VP1 staining (green only), indicating a large fraction of internalized virus (Fig 4C). B4St8 ganglioside KO-MEFs also displayed high levels of internalized virus as shown by line scan analysis (green only) (Fig 5C). These data demonstrate that gangliosides are not required for virus entry into MEFs, and virus can enter cells through non-ganglioside mediated pathways. Given the complete resistance of these B4St8 KO cells to MuPyV infection (Table 1), it can be assumed that virus uptake *via* these alternative routes proceeds along a non-infectious or ‘dead end’ pathway. While the presence of glycoproteins such as α4β1 integrin have been observed to enhance infection [18, 19], gangliosides are required for virus uptake along infectious pathways.

### Fibroblasts from ganglioside-deficient mice show a normal mitogenic response to the virus

**Fig 6:**
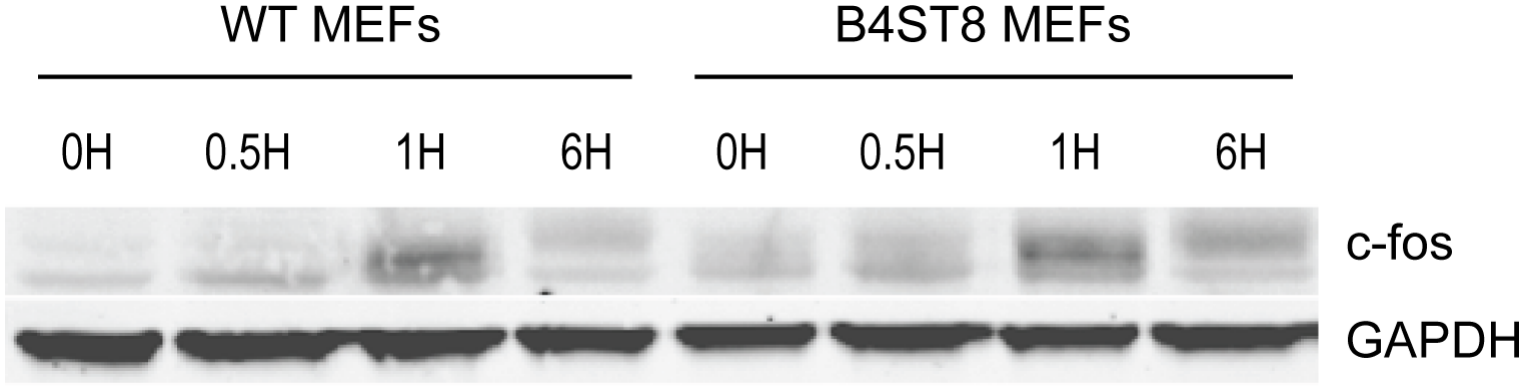
Induction of c-fos in wild-type and B4St8 KO MEFs. Purified virus in serum-free medium was used to infect wild-type and B4St8 KO MEFs. Cell extracts were made at the indicated times post-infection and subjected to western blot analysis with anti-fos antibody.

An earlier study demonstrated the ability of MuPyV to induce expression of *c-fos* and other ‘early response’ genes in established mouse fibroblasts [30]. This response, measured by mRNA synthesis, is biphasic. A rapid but transient response occurs within the first hour followed by a second sustained wave of expression beginning around 12 hrs post-infection. The second wave requires early viral gene expression, while the initial transient phase can be induced by empty capsids or recombinant VP1. To determine if the induction of an immediate early response depends on recognition of gangliosides or other virus receptors, wild-type and B4St8 KO MEFs were exposed to purified virus in serum-free medium (Fig 6). Extracts were analyzed for c-fos protein by western blot. The double KO MEFs responded indistinguishably from wild-type MEFs with a clear induction at 1 hr post-infection. At 6 hrs post-infection, the response was diminished but showed the slower migrating form(s) of the c-fos protein indicative of phosphorylation which is known to accompany activation of mitogen receptors (Fig 6) [31]. We conclude that the rapid induction of c-fos in ganglioside-deficient cells results from virus binding to non-ganglioside receptor(s).

### Splenocytes from ganglioside-deficient mice show a normal innate immune response to the virus

Antigen-presenting cells from mice that are naturally resistant to tumor induction by MuPyV respond to the virus by secretion of IL-12, a type 1 cytokine [32, 33]. This IL-12 response does not require infection and can be elicited by exposure to virus-like particles assembled from recombinant VP1. Pretreatment of splenocytes with neuraminidase prevents the IL-12 response, consistent with roles of gangliosides or glycoprotein, or both, as receptors mediating the innate immune response [33].

TLR4, a known sialoglycoprotein, has previously been implicated in mediating cytokine responses to MuPyV. Quantitave Trait Locus analysis of an F2 cross between susceptible and resistant mice pinpointed a region of chromosome 4 encompassing TLR4 as the determinant of the cytokine response. Transfection of macrophages from a TLR2/TLR4 double KO mouse with TLR4 cDNA from a resistant strain conferred an IL-12 response. While pointing clearly to a role of TLR4, these results do not rule out the possibility that gangliosides may play an essential or supporting role in mediating the IL-12 response. We therefore investigated the immune response in the absence of ganglioside receptors.

B4 KO and St8 KO mice were derived on a background of the resistant C57BL/6 strain that responds to MuPyV with IL-12. To determine if this innate immune response is retained in ganglioside-deficient mice, splenocytes were harvested from naive wild-type, B4 KO and St8 KO mice. Cells were exposed to virus for 1 hr and incubated further for 40 hrs. Culture supernatants were then assayed for IL-12 by ELISA. Splenocytes from all three strains responded to the virus by secretion of varying levels of IL-12 indicating that gangliosides are not required for the IL-12 response (Table 3). Interestingly, splenocytes from B4 KO mice showed the strongest response, roughly 2-fold greater than the wild-type, indicating that gangliosides may dampen the IL-12 response possibly through competition with TLR4 for virus binding. Pre-treatment of cells with neuraminidase from *Vibrio cholera* which cleaves sialic acids from both glycolipids and glycoproteins completely inhibited the response, as expected. Importantly, pretreatment with PNGase F, an endoglycosidase that cleaves oligosaccharide chains from N-linked glycoproteins, also inhibited the response. These results demonstrate that gangliosides are not required for the innate immune response to MuPyV, and ganglioside-deficient cells have a heightened IL-12 response upon virus challenge compared to wild-type and St8 (GD1a and GT1a containing) cells.

**Table 3:** IL-12 response to MuPyV by splenocytes in KO mice.

Cells	Untreated	Neurase	PNGase
Mouse Wild Type	3105 ± 90	<25	405 ± 13
ST8 KO	3150 ± 58	<25	341 ± 59
B4 KO	6025±272	<25	400 ± 1

Five million spleen cells were pretreated with either Neuraminidase (Neurase) (400 units/mL) or PNGase (10,000 units/mL). Cells were exposed to MuPyV and cultured for 40 hr. Supernatants were tested for IL-12 by ELISA (pg/ml). Cells stimulated with medium alone gave <25 pg/ml. Shown are the mean ± SD of four 4–5 week old mice for each determination.

## Discussion

Ganglioside supplementation experiments in ganglioside-deficient rat and mouse cell lines have identified specific gangliosides (GD1a and GT1b) as MuPyV receptors; however, these findings have not previously been validated *in vivo*. Additionally, the role of gangliosides in other host-responses to MuPyV infection such as mitogenic gene induction and innate immunity has not been investigated. We generated mouse strains that are deficient (St8 KO) and null (B4 KO) for complex gangliosides to further characterize the specificity of ganglioside-mediated host responses to MuPyV *in vivo*. The St8 KO mice are deficient in GD3 synthase and do not synthesize b-series gangliosides (GD1b and GT1b) but retain synthesis of the a-series gangliosides (GD1a and GT1a) (Fig 1A and 1C). St8 KO mice succumb to LID infection, indicating that a-series gangliosides are sufficient to mediate a lethal virus infection. (Fig 2A). The B4 KO mouse lacks GM2/GD2 synthase, which is required for both a-series and b-series ganglioside synthesis (Fig 1A and 1C). Our finding that newborn B4 KO mice are completely resistant to infection by the normally lethal LID strain of MuPyV provides clear evidence that gangliosides are required for infection and spread of the virus in the animal. The LID strain was chosen to evaluate the role of gangliosides in the animal because of its rapid effects ending in death as a discrete endpoint. As the KO strains derive from tumor-resistant C57BL/6 mice, the PTA and RA strains cannot be evaluated for their ganglioside dependence in a tumorigenic setting using these mice. However, based on the fact that these strains of MuPyV, like LID, are unable to infect B4St8 KO MEFs, it is expected that they would be unable to infect ganglioside-deficient mice. Results using LID have shown for the first time that the GM2/GD2 synthase pathway is necessary and sufficient for MuPyV infection *in vivo*. This finding in the natural host is an important *in vivo* validation of earlier biochemical and cell culture results [2, 28, 29]. Additionally, these mice provide an excellent model to test the role of gangliosides in other host responses to MuPyV infection.

Early biochemical and *in vitro* experiments were extremely valuable in first identifying gangliosides as possible receptors for MuPyV based on ganglioside binding to VP1-pentamers [2, 28, 29]. However, results presented here have shown that distinctions must be made between gangliosides that bind *in vitro* and those that operate as functional receptors *in vivo*. GD3 was identified in a screen of a glycan array as an effective binder of VP1-pentamers. The GD3 glycan also binds recombinant VP1 pentamers in crystallographic studies [26]. This ganglioside however does not confer susceptibility to infection under normal conditions (B4 KO, Fig 2A). The observation that B4 KO mice have high levels of GD3 in the kidney (Fig 1B), yet are resistant to infection, is convincing evidence that this ganglioside is unable to mediate infection *in vivo* despite binding to VP1 *in vitro*. Discrepancies between results of biochemical binding and *in vivo* infection are important to recognize for a full evaluation of receptors from a functional standpoint.

Previous experiments have shown that GD1a or GT1b are sufficient for infection of ganglioside-deficient rat glioma cells [2, 28]. We identified additional gangliosides, the a-series ganglioside GT1a and the b-series ganglioside GD1b, as receptors for RA MuPyV. When fibroblasts from B4St8 ganglioside-deficient mice were pre-incubated with gangliosides GT1a or GD1b their infectibility was restored (Fig 5). We also confirmed that GD1a as a receptor for MuPyV in the ganglioside-deficient B4St8 MEFs. Because B4St8 MEFs lack all complex gangliosides, these data show that supplementation with single gangliosides is sufficient for MuPyV infection.

Structural studies of the MuPyV capsid protein VP1 have revealed that VP1 binds sialic acid within a pre-formed sialic acid binding pocket on the virus surface [10,11]. These results suggest that MuPyV could potentially bind sialyated oligosaccharides on either glycoproteins or glycolipids (*i*.*e*., gangliosides) through interactions with sialic acid. Thus, we evaluated MuPyV cell surface binding in the presence or absence of ganglioside receptors. We found that while virus binds to the cell surface of B4St8 KO MEFs, it accumulates to lower levels than in wild-type MEFs or GD1a-supplemented B4St8 MEFs. These results suggest that virus binding to gangliosides is required for high levels of virus accumulation on the cell surface, although the presence of glycoprotein or other interactions on ganglioside-deficient cells allows for some virus binding to the cell surface. Cell surface binding of virus is not necessarily indicative of virus entry. Therefore we determined if virus enters cells in the absence of ganglioside receptors. We observed that MuPyV is internalized in the absence of gangliosides. Importantly, uptake of virus under these conditions does not lead to infection. Previous studies have shown that proteinase treatment of cells prior to MuPyV addition leads to slight increases in infection, suggesting that MuPyV-glycoprotein interactions inhibition MuPyV infection [34]. This inhibition may be due to altered trafficking of MuPyV, whereby ganglioside receptors mediate transport of MuPyV to the ER along an infectious pathway while glycoproteins act as “decoy receptors” and lead to MuPyV degradation [34]. Our results are consistent with the idea that MuPyV-glycoprotein interactions lead to non-infectious pathways of entry. It is possible that glycoprotein interactions mediate other host responses to virus infection, such as mitogenic signaling, or the immune response. Alterations of these responses are not readily apparent in infection-based cell culture experiments.

In order to understand how host responses change when ganglioside interactions are lost we evaluated two known cellular responses to MuPyV infection in the B4St8 KO cells: a) activation of mitogenic gene induction, and b) activation of innate immune responses. Fibroblasts from B4St8 KO MEFs responded to MuPyV binding with a rapid transient induction of *c-fos* that is indistinguishable from the response of wild-type MEFs. Thus gangliosides are not required for mitogenic gene induction by MuPyV and this response may be triggered by virus binding to any of a number of sialoglycoproteins (or other molecules) that normally serve as mitogen receptors in the plasma membrane. These include receptors for platelet-derived growth factor, epidermal growth factor, insulin-like growth factor, and others, whose activation leads to cell cycle progression and entry of cells into S phase essential for facilitating the initial replication of viral DNA [30].

It has previously been shown that antigen-presenting cells from wild-type mice that mount effective adaptive anti-tumor responses respond to virus challenge at the innate level by secretion of the type 1 cytokine IL-12. TLR-4 is required for the IL-12 response in these mice and mice lacking TLR-4 are suspectible to MuPyV tumor induction, likely due to a loss of IL-12 secretion [34]. MuPyV is thought to bind to TLR-4 through sialic acid containing oligosacharride regions on the extracellular domains of TLR-4. To confirm that sialic acid interactions are required for IL-12 induction we treated splenocytes with neuraminidase prior to challenge with virus. As expected, pretreatment with neuraminidase blocks the cytokine response to MuPyV in both wild-type, St8 KO, and B4 KO cells; however, neuraminidase treatment also abrogrates MuPyV-ganglioside interactions and thus is not informative about the role of gangliosides in IL-12 induction. We sought to abolish MuPyV-TLR4 interactions while retaining ganglioside interactions, by treating with PNGase, which removes N-linked carbohydrate chains from glycoproteins, prior to virus challenge. We found inhibition of the IL-12 response by pretreatment with PNGase providing further support for TLR4 as a required receptor in the innate immune response to MuPyV and confirming that MuPyV-ganglioside interactions are not sufficient for IL-12 induction. Lastly, we wanted to determine whether gangliosides contribute positively or negatively to the innate immune response induced by virus binding. Splenocytes from B4 KO mice display an increased IL-12 response compared to wild-type or St8 KO splenocytes. These data indicate that gangliosides may dampen the cytokine response, possibly by competing for virus binding with the TLR4 glycoprotein receptor. This observation suggests that glycolipids and glycoprotein receptors act in an opposing manner in multiple ways, even at the level of the innate immune response.

Gangliosides contribute to a diverse array of physiological responses involved in viral infection. Results of experiments in ganglioside-deficient mice show that while gangliosides are essential as receptors for MuPyV infection, they are not essential for cell surface binding, cell entry, or for activating the early mitogenic and innate immune responses of the host. Additionally, the antiviral immune response was heightened in ganglioside-deficient splenocytes, indicating that gangliosides somehow serve to dampen the antiviral cytokine response. These data establish that multiple types of receptors bearing sialic acid are utilized by the virus to mediate different aspects of virus-host interaction. These results could have implications for tissue tropism and immune response generated *in vivo* by other Polyomaviruses.

## Materials and Methods

### Ethics statement

This study was carried out in strict accordance with the recommendations in the Guide for the Care and Use of Laboratory Animals of the National Institutes of Health. The protocols were approved by the Harvard Medical Area Standing Committee on Animals (approval numbers 298 and 781). All mouse strains were maintained in specific pathogen-free conditions in the animal facilities of the Harvard Medical School. All efforts were made to minimize suffering and provide humane treatment to the animals included in the study.

### Ganglioside-deficient mice

Two knockout strains of mice were obtained through the Mutant Mouse Regional Resource Center, Missouri/Harlan Consortium, by cryo-resuscitation and embryo transfer. Strain B6;129S-*B4galnt1* ^*tm1Rlp*^/Mmmh [Stock #: MMRRC:000036-MU] is a knockout in the B4galnact1 (beta-1,4-N-acetyl-galactosaminyl transferase 1) gene. It is referred to here as the B4 KO. B4 -/- KO mice are male sterile; the colony was maintained by crossing heterozygous B4 +/- males with B4 -/- or B4 +/- females. Strain B6;129S-*St8sia1* ^*tm1Rlp*^/Mmmh [Stock #: MMRRC:000037-MU] is a knockout in the St8sia1 (ST8 alpha-N-acetyl-neuraminide alpha-2,8-sialyltransferase 1) gene. It is referred to here as the St8 KO. A double knockout mouse, B4St8 KO, was generated by crossing B4 KO and St8 KO mice. Genotyping was carried out on tail DNA by PCR using the suppliers protocols.

### Ganglioside analysis

Solvent A—isopropanol-hexane-water (55:25:20, v/v/v; upper phase discarded)
Solvent B—chloroform-methanol (1:1, v/v)
Solvent C—chloroform-methanol-water (30:60:8, v/v/v)
Solvent D—chloroform-methanol-water (50:40:10, v/v/v) + 0.03% (w/v) CaCl_2_

Crude gangliosides were extracted from kidneys of wild-type and B4 KO mice by homogenization in 20 mL solvent B with a Polytron homogenizer. The solution was centrifuged, the supernatant transferred and the homogenization repeated two times with 20 mL solvent A and once more with 20 mL solvent B. The four extracts were pooled and dried on a rotary evaporator. Gangliosides were purified from the crude fraction by DEAE-Sephadex Ion Exchange chromatography. The crude fraction was resuspended in a minimal amount of solvent C and applied to a column of DEAE-Sephadex A-25 (acetate form). Neutral species were eluted with 5 volumes of solvent C. Acidic species were eluted with 5 volumes of 0.5 M sodium acetate in methanol. The acidic fraction was dried, dialyzed exhaustively using a Pierce Slide-a-lyzer cartridge (MWCO = 3500) in deionized water and redried. Neutral and acidic fractions were dried and taken up in Solvent A for HPTLC analysis.

Analytical high-performance thin-layer chromatography (HPTLC) was performed on silica gel 60 plates (E. Merck, Darmstadt, Germany) using solvent D as the mobile phase. Lipid samples were dissolved in solvent B and applied by streaking from 5 ml Micro-caps (Drummond, Broomall, PA). Detection was with Bial’s orcinol reagent [0.55% (w/v) orcinol and 5.5% (v/v) H2SO4 in ethanol-water (9:1, v/v); the plate was sprayed and heated briefly to ~200–250°C.

### MuPyV virus strains

The PTA and RA strains have been described (CD—AJP, 1987; RF, GMDubensky 1991; Freund, AC 1991; CD,RF 1987). PTA is a standard large plaque ‘high tumor’ strain that binds straight chain oligosaccharides with terminal α 2,3-linked sialic acid. RA is a standard small plaque ‘low tumor’ strain that binds branched as well as straight chain sialic acids [23, 24, 35]. LID is a virulent strain derived from PTA [23, 27]. All strains were propagated in primary baby mouse kidney cells.

### Infection of ganglioside-deficient mice

Newborn WT, B4 (-/-), B4 (+/-) and St8 (-/-) mice were inoculated intraperitoneally within 24 hrs of birth with 1–2 x 10^6^ plaque forming units of the LID strain of virus. Mice were followed on a daily basis and results recorded based on death as an endpoint. Mice were genotyped retrospectively, *i*.*e*., after death or at the termination of the experiment at 35 days.

### Infections of cells from ganglioside-deficient mice and immunofluorescence assay for large t antigen expression

Fibroblast cultures (MEFs) were prepared from embryos of 18 to 19 days gestation and genotyped to generate B4 KO, St8 KO and B4St8 KO mouse embryo fibroblast lines (MEFs). MEFs were maintained by serial passage in Dulbecco’s Modified Eagle’s medium with 10% fetal bovine serum and used for viral infections at passages between 2 and 5. Cells on coverslips were infected by MuPyV strains RA or PTA at various multiplicities of infection and fixed at 24 hrs post-infection with 4% neutral buffered paraformaldehyde (Electron Microscopy Sciences, Ft Washington, PA). Cells were permeabilized with 0.3% Triton X-100 in PBS and stained with rat polyclonal anti-T antibody (Goldman & Benjamin 1975) and rhodamine-conjugated donkey anti-rat IgG.

### Confocal microscopy for virus entry

WT and B4St8 MEFs were seeded onto glass coverslips in Dulbecco's Modified Eagle's Medium supplemented with 10% fetal bovine serum (FBS). Cells were incubated overnight in serum free media prior to infection. Cells were then infected with RA MuPyV (MOI 50). At indicated times post infection (30 min, 3 hrs) cells were washed in phosphate buffered saline (PBS) and fixed with 4% paraformaldehyde (PFA) at room temperature (RT) for 10 mins. Cells were blocked in 10% FBS in PBS overnight at 4°C followed by staining for cell surface VP1 (I58 antibody/Alexa Flour secondary 546). Cells were then re-fixed with 4% PFA at RT for 10 mins followed by permeabilization with 0.5% Triton X-100 for 15 mins at RT. Cells were blocked in 10% FBS in PBS overnight at 4°C followed by staining for total VP1 (I58 antibody/Alexa Flour secondary 488). Confocal images were taken as a 5 step (.125 μm step size) z-stack and slices were taken through the center of the cells. Each z-stack was aligned and compressed into a max intensity Z projection image for quantification of cell surface and total VP1 staining. Using the Nikon software, line scans were taken sampling the cell surface and cytoplasm of each cell to measure both cell surface and internalized virus as indicated by VP1 staining.

### Immunofluorescence

Cells were seeded onto glass coverslips in Dulbecco's Modified Eagle's Medium supplemented with 10% fetal bovine serum (FBS). Cells were incubated overnight in serum free media prior to infection. For ganglioside-supplemented cells, serum free media containing the indicated concentration of gangliosides was used. Cells were then infected with RA (MOI 5 to 10). At indicated times post infection cells were washed in phosphate buffered saline and fixed with 4% paraformaldehyde at room temperature (RT). Cells were blocked in 10% FBS and then stained for GD1a using the MAB5606 (Millipore). Samples were then incubated with Alexa Fluor labeled secondary antibodies. Cells were imaged on a Nikon A1R confocal microscope.

### Image analysis

Confocal images were taken as a 9 to 13 step (.25 μm) z-stack. Each z-stack was aligned and compressed into a max intensity Z projection image for quantification of T-antigen staining. To quantify infection, T-ag staining was measured per each DAPI labeled nuclei. The DAPI channel on each image was thresholded and nuclei were counted using ImageJ (Analyze Particles). These particles were marked as “Regions of Interest” (ROI) and then the average pixel intensity of T-ag staining was measured for each nuclei (ROI). These were then binned into T-ag positive or T-ag negative nuclei to determine % infected.

### Flow cytometry

Cells were dissociated from the plate with Versene solution (EDTA) for 10 mins at room temperature (RT). Resuspended cells were then washed in cold PBS followed by incubation with MuPyV (RA, MOI 10) on ice. Samples were removed at indicated time points and temperatures, washed with cold PBS, and fixed with 0.5% paraformaldehyde (RT for 5 mins), followed by staining for VP1 (I58). Cells were not permeabilized. Cell surface virus levels were measured for >10,000 cells per sample by flow cytometry using a CyAN ADP Analyzer.

### C-fos induction assay

Wild-type and B4St8 MEFs were plated at 3 x 10^5^ cells per 35 mm plastic tissue culture dish in DMEM with 10% fetal bovine serum. Twenty-four hrs after plating, the plates were washed and the medium replaced by DMEM with 0.1% platelet-poor plasma for an additional 12 hrs prior to infection. Infection was carried out with purified virus in serum-free PBS buffer at a MOI of 20 to 40 PFU/cell. Virus was purified by cesium chloride density gradient centrifugation as described [36, 37]. Cells were incubated for 1, 2 and 6 hrs post-infection in serum-free DMEM. Total cell proteins were extracted, separated by SDS-PAGE and blotted for c-fos protein with antibody from Santa Cruz Biochemicals.

### IL-12 response in splenocytes

Assays were carried out as described [38]. Briefly, five million splenocytes were harvested from naïve 4–5 week old wild-type B6, St8 KO and B4 KO mice. 5 x 10^6^ cells were exposed to MuPyV RA strain at an MOI of 2 to 5. Culture supernatants were harvested after 40 hrs and levels of IL-12 determined by ELISA. Determinations were carried out on four mice of each strain. Measurements were also made on cells pretreated with neuraminidase from *V*. *cholera* (400 Units/ml) or by endoglycosidase PNGase F (10,000 Units/ml) (Calbiochem) prior to infection.
